# The New York Institute of Stomatology

**Published:** 1901-01

**Authors:** 

**Affiliations:** The New York Institute of Stomatology


### Reports of Society Meetings.

### THE NEW YORK INSTITUTE OF STOMATOLOGY.

A REGULAR meeting of The New York Institute of Stomatology was held Tuesday evening, October 2, 1900, at the office of Dr. S. E. Davenport, 51 West Forty-seventh Street, Dr. C. O. Kimball, of the Executive Committee, in the chair.

The minutes of the previous meeting were read and approved.

### COMMUNICATIONS ON THEORY AND PRACTICE.

Dr. H. S. Bascom read a paper entitled "Case of Irregularity of Superior Cuspid."

(For Dr. Bascom's paper, see page 17.)

*Dr. Kimball.*—It is very unsatisfactory to get hold of a piece of work and yet be obliged to leave it unfinished, and I think our sympathies and hopes are all with Dr. Bascom that he will succeed in completing the work he has so well commenced.

Will you allow me to bring to your attention a case of my own which this suggests? I have the advantage of Dr. Bascom, however, in that my case is completed, and I have my patient here to show. The history is a simple one. Nos. 1 and 2 show the models of the mouth as it came to me. The young man was nearly eighteen years old. The lower arch was in good shape. The upper jaw was much twisted, so that the arch was not only crowded, but with nearly a reversed curve on one side. No trace whatever of the right upper permanent canine; the temporary canine was still in its place.

A careful examination of the mouth, as will appear on the model, showed a slight prominence (indicated by a blue pencil mark on the model) in the roof of the mouth below the lateral incisor. On feeling high up outside, I imagined that I could locate the tip of the root of the tooth as it lay embedded in the jaw. I have indicated by blue pencil where the point of the crown and the tip end of the tooth seemed to be. It was so high up that it was a doubtful problem whether the tooth was there or not. After consultation with various friends I made an exploratory incision, and found that I was right. The tooth was there.

The tooth was left alone the first summer, the time being spent in broadening the greatly contracted arch as a preliminary operation. In the fall of 1899 the prominence on the inside of the mouth had become a little more marked than was the condition on the 5th of November, 1898.

After broadening the arch the first thing to be done was to spread the teeth apart on the right side by means of wedges, to allow the tooth space to come down in place. We wedged between the bicuspid and the temporary canine, using the latter simply as a block. Having space, we made a clean incision to the tooth and packed the gum back till it healed, leaving a pit with the point of the canine at the bottom. I then made a little catch at the corner of the tooth and put on this appliance No. 3, a Jackson plate, with a little spring with a sharp point to catch the tip of the tooth and draw it into position. In the course of three months that had worked right out, and we have it in this shape where the tooth begins to show here in very nearly its proper position. The next mould is missing. It shows the tooth fully brought down, but twisted. A band was fitted to the tooth and a spring attached to the plate, hooking into a little eye on the band. During the following season the other teeth were straightened. This model shows the position of affairs as it is now.

I have asked the young gentleman to come here this evening (showing patient). He still wears the retaining plate with the spring hooked to the band; the tooth has been twisted nearly one-quarter of a turn.

The date of commencement was in June, 1898. We spent the first summer in simply broadening the arch, the winter in getting the tooth out into position, the spring in twisting it, the past summer in regulating the teeth.

*Dr. S. E. Davenport.*—Dr. William H. Jones, of Northampton, Mass., has just sent me several mouth-mirrors, recently designed by him, with the request that they be exhibited to the Institute. He calls them "Jones sterilizable mouth-mirrors," as they can be put in boiling water without injury, the mirror being of highly polished metal instead of glass. They are screwed into a simple aluminum handle and are interchangeable. They are of several shapes; some magnify and some are plain.

Dr. Jones does not intend to patent the mirrors, I understand, but they will probably be on the market in due time should there be a demand for them. He does not claim that they possess any special advantage over glass mirrors, except that they can be sterilized without injury and are non-breakable.

*Dr. Kimball.*—Dr. A. F. Davenport, of North Adams, Mass., will now speak to us on "Stray Thoughts on Dental Hygiene."

(For Dr. Davenport's paper, see page 22.)

*Dr. E. H. Raymond.*—The subject that has been presented to us this evening is one that interests us all. Our first duty in treating diseases of the mouth is to put the mouth in a thoroughly aseptic condition. If we can counteract the solvent action of acids and destroy micro-organisms, normality can be restored in the oral cavity. To keep it so is the problem. Although this is a progressive age, and we have at command many remedial agents that patients can use, it is a difficult thing to get them to help themselves with anything except a tooth-brush. They are slow to adopt other prophylactic measures. How often, after we have toiled to put their mouths in proper condition, and given careful instructions, have we seen them return in a few months and find that no effort on their part has been made to help themselves except to brush the labial surfaces of their teeth.

It is my custom to prescribe hydrogen dioxide and milk of magnesia whenever I see any tendency to pyorrhœa or cervical decay. I believe that a three per cent. solution of hydrogen dioxide (aqueous), used daily, to be the best and safest preparation that a patient can use for all infectious conditions of the mouth. It is a good antiseptic and free from unpleasant taste. As an antacid I prefer the milk of magnesia. This should be freely used just before retiring, especially around the necks of the teeth.

Let us remember that however vigilant we may be during the daytime, the "night cometh" when acids get in their solvent work and the microbe is ever busy in the process of demolition.

*Dr. S. E. Davenport.*—Dr. A. F. Davenport's paper bears pertinently upon many of the causes of dental caries and outlines good ideas, which, if followed, would result in the prevention of much tooth destruction.

For some years I have taken the position, in conversation with my patients, that the teeth of a person in good health will be almost free from decay if food is not allowed to remain between and about them. This freedom from putrefying material can be gained only by the thorough and scientific use of the brush and floss silk after each meal at least, and the thorough disinfection of all contiguous mucous surfaces.

A considerable part of a dentist's duty is the instruction of patients in the proper use of the brush and silk, not only that the entire tooth surface may be polished three or four times a day, but that the gum be thoroughly massaged as well,—a most important point,—and the brush so directed that recession of the gums will be prevented rather than encouraged.

*Dr. J. F. P. Hodson.*—I do not find so much difficulty in having my patients brush their teeth as in having them use the silk floss. I am continually saying to them that if they are going to give up either the use of the silk floss or the brush, by all means give up the brush. The constant friction of the lips, tongue, and cheeks and the flow of saliva tend to cleanse the surface of the teeth, while the action of the brush tends to sweep the deposits in between the teeth, where decay is most apt to occur, the place which needs the most care and receives the least. In the use of the brush, however, I am careful to suggest that they brush off the tongue as well as the teeth. It seems to me very foolish to take so great care to brush the teeth thoroughly and leave a very dirty tongue to immediately soil them again. With the many aids we have now as compared to what we used to, it would seem that we ought to be showing good results from our constant teachings.

I can hardly agree with one of the speakers in regard to milk of magnesia. I have been disappointed that it would not do the work I hoped it would to render the mouth antacid. I did not find that it did so. I have thought perhaps the glycerin in it gave an acid reaction. I returned to my precipitated chalk, and urge my patients to rub it upon the gums and between the teeth just before they retire at night.

*Dr. L. C. Leroy.*—I think that Dr. Davenport will agree that the key-note of much of our trouble lies in the fact that we have to go farther back than attention to the teeth, and that we must adopt positive measures.

My experience with milk of magnesia, applied externally, has not been as efficacious as it should have been, but when administered as an internal remedy it has acted much better.

I have in mind a patient, a young man, who had serious erosion of the lingual surface of the incisors, where the lower incisors came in contact with the upper. I feared very serious results for the young man's teeth. Internal use of milk of magnesia was prescribed, taking a teaspoonful at night, permitting the precipitate to form upon the teeth before swallowing. I know of no other reason why the erosion should have ceased, but it did so, which has inclined me to think that if we would go farther than dentists usually go, putting our patients in good physical condition generally, and bringing into effect medical skill, we would see far better results. If one feels that he is not competent to prescribe generally, he should refer patients to their family physician. There are many cases which might require general medical treatment; in pyorrhœa, for example.

Dr. Davenport seemed to lay particular stress upon the instruction in schools in relation to the care of the mouth. That I think is paramount. The doctor asked what has been done in New York City. I think that there has been a course of lectures instituted in our public schools and some of our high schools. A few men—those who lecture in the schools—make it a practice to examine some mouths in the districts in which they reside. We are in hopes that some good will result in the future, but I suppose that we must be content with what we have so long as the majority of our profession do not care to put their shoulders to the wheel and give their services gratuitously for the care of the suffering poor.

*Dr. C. F. Allan.*—The able essay that has just been read is one that must impress itself favorably on every one present, for the subject treated is of the first importance. Prophylaxis should be our highest aim, but, following the argument of the essayist, I cannot see how, except in the most general way, the care of the teeth can be taught in the public schools. One patient comes to the dentist with teeth irregular in arrangement and with mucus so thick that it can be strung out by the yard. Now, that patient requires every aid available for the proper sanitation of his mouth; while another patient with teeth in perfect arrangement and secretions thin and limpid, with the gums hard and practically no tendency to pyorrhœa, can keep his teeth in good order with the minimum amount of attention, none at all almost as compared with the case first mentioned.

The dentist who has these individual cases before him can instruct such patients intelligently how to look after their teeth, but I do not see how the special attention necessary can be indicated from the rostrum of a school-room. The main thing is that the teeth must be kept clean, and I impress upon my patients that it lies with them to see that they are kept clean, and to know when they are well cleansed, just as they are to know when their hands and faces are clean,—by examination. Of course it will have to be done with the aid of a mirror, possibly a mouth-mirror, but they must actually *see* that their teeth are clean.

When the patients are in the chair call their attention to the purple color of the gum around a lower cuspid, and tell them what it indicates, warn them that free bleeding of the gums, as a rule, indicates pyorrhœal conditions that could have been prevented, and so impress upon them the proper look of a healthy and cleanly mouth that, examining it before a glass, they cannot be mistaken. A hard and fast rule that shall tell all people how to take care of their teeth does not exist and cannot exist.

*Dr. Kimball.*—It would seem as if this subject were spoken of so many times that it would be quite exhausted, but we know that it is not. It is always fresh in our minds. I have been surprised to hear people say they cleaned their teeth once a day, and on asking them, "When do you do it?" the reply would often be, "Before breakfast." "Well," I used to say, using the same illustration that Dr. Davenport has spoken of, "suppose you were in the habit of going among your plants, would you be satisfied if you washed your hands once a day, and that before you potted your plants?" bringing up the point that Dr. Davenport has,—to clean our teeth when they are soiled, and to keep them clean, that is all. There we have the hygiene in a nutshell.

*Dr. A. F. Davenport.*—I have noticed among most of my patients that the injury has been done largely before I saw the patient. My idea is that if we could by some means get the children to form the habit of caring for their teeth early it would be a great advantage to them as well as to the dentists. My idea in having the habit of caring for the mouth and teeth established in the schools is to reach a class that we seldom see, and by so doing to accomplish as much good to that class as possible.

*Dr. Kimball.*—We will next listen to Dr. T. W. Onderdonk, who will describe his treatment of unfilled pulp-canals.

(For Dr. Onderdonk's paper, see page 20.)

*Dr. F. Milton Smith.*—I have listened with a great deal of interest to Dr. Onderdonk. I could not help thinking, while he was reading, that if I could write and get to the point as promptly, and sit down, I should be a vast improvement on myself. If there is but one point out of six in which Dr. Onderdonk differs from me, he agrees with me better than my friends usually do. They usually think me nine-tenths wrong and one-tenth right. If I am getting to be five-sixths right, I am making an improvement.

I think I said, in the paper referred to, "When I get it to an aseptic condition, I immediately fill the root." If I understand the doctor aright, he thinks there may be some question as to when I got there, and I suppose there are many times when I have failed in getting there. I have not a bacteriologist in my office to see when the root is ready to fill. If I had I might ask him when it was time. Thus far, after having followed the four or five conditions laid down in the paper, I have almost invariably found that the filling has not required removal. When the doctor was reading his paper I thought of a confession to make. In reading my paper I stated that I did not believe that in the twelve or fourteen years I had practised the method I had lost three teeth. Not two weeks after I had read that paper I had a young lady patient in whose tooth some one had kindly used arsenic. I treated and filled it, but the suffering was so intense that I sent her to Dr. Hasbrook, who extracted the tooth. I had requested her to secure the tooth for me. When I received it I cut off the crown from the root, and felt carefully for the filling, and found that the root was unfilled at least one-third from the apex. If that goes to prove anything, it is that trouble is more likely to ensue if the root is not well filled.

*Dr. S. E. Davenport.*—Dr. Onderdonk's paper is a practical statement of facts from experience, and we plainly see that he is accustomed to the filling of roots. He speaks feelingly, and I judge that there have been some exciting times in his practice which have taught him when *not* to fill roots at the first sitting.

While the subject chosen by Dr. Onderdonk is a very old one, there is still much to be learned about it, and it is well for us all to hear a clearly defined paper like this, as our thoughts are thereby turned in the right direction.

*Dr. G. S. Allan.*—The philosophy of the management of so-called dead teeth is simple enough, but working out the details and putting into practice the laws and principles that govern the management is quite another matter. A large knowledge of science in general is necessary to enable one to work intelligently, and an equal amount of mechanical skill and dexterity to put them into practice.

The subject easily divides itself into two parts,—(1) the preparation of the pulp-canal, and (2) the closing up and filling of the same. The two parts are of equal importance, but not necessarily of equal completeness of detail in practice. Were it so, our list of failures would largely exceed our successes. Complete sterilization of the canal is most essential, but a perfect root-filling—one closing the foramen with exactness, not going beyond and leaving no vacant spaces in the canal—may be, and frequently is, dispensed with and yet success crown one's efforts.

The ideal operation is the one that destroys all inflammatory pus-forming germs in the canals and the parts adjacent to the foramen and then prevents their obtaining a future lodgement from without. No amount of skill or forethought can prevail against the germs that wander through the system and do their evil work whenever and wherever they obtain a lodgement in a favorable soil. Fortunately this latter danger does not often worry us or give suffering patients a cause for complaint. There was a time when I fairly dreaded to see a case where any of the temporary teeth required the removal of the pulp and their treatment. Now they do not disturb me. Formerly I thought it necessary to not only remove the dead pulp, but to fill the empty roots. I had been educated to believe that radical root-filling was a *sine qua non,* and so endeavored to put my practice on an equal footing with both sets of teeth. Now that I am able to see more clearly what must and what need not be done, my dread has departed and my success is more certain.

Thought and study of results and causes have taught me that it is not only safe to leave the roots of the temporary teeth open, but that it is the wisest and safest practice, and carrying out the principles involved to their logical conclusion. I now do not hesitate to leave undone that which I cannot do, and leave unfilled, in part, roots where from their nature and shape I cannot attain the ideal thing. The conclusion is that a root sterilized in all its parts is practically safe even if it is not up to the highest standard so far as the filling is concerned. A professional friend in a neighboring city once told me that he seldom filled any roots,—did not think it necessary,—and yet he was an able man, had a large practice, and courted success with high standards.

How often have we all seen cases where the pulp has died and been allowed to remain in the pulp-chamber and roots for years and given no pain or trouble. So long as germs were excluded they would remain quiet. The dead pulp, though an excellent soil for the propagation of germs, was harmless till they were introduced.

Nothing that I have so far said must be interpreted to mean that I do not advocate thorough root-filling. I most certainly do, but I sadly recognize my limitations and am ready to stop when I have done my best, and still hope for a fair amount of success though I am compelled to stop short of the ideal. Therefore it is that I say, look to the removal of the dead pulp and the thorough sterilization of the roots first, and be thankful that so much can be done to a finish.

Hydrogen dioxide is by far the most satisfactory germicide in many ways, but the free oxygen liberated is apt by its mechanical pressure to prove irritating and give much pain. In potassium permanganate we have an excellent substitute and one free from the objectionable features of the hydrogen dioxide.

It may be well to state in brief the real value of root-filling. As a matter of fact, whether the operator fill the root-canal or not, the space once occupied by the pulp will be filled by something. It cannot remain vacant. In some way organic matter will find an entrance into the open root, and lifeless organic matter at that, and therefore just the right soil for the pathogenic germs to flourish in. The rationale of root-filling, then, is not so much that the germs may be kept out of the roots as that the food on which they grow may be kept away from them. We plan to render them harmless by starving them. Once started growing, they make their own pabulum by destroying some of the adjacent tissue. No matter how disproportionate a part of the apical portion of the root-canal remains unfilled, if any portion remains outside the life forces a focus for the active work of the germs is ready at hand. The germs we fear are those that obtain an entrance to the pulp-chamber from the oral cavity.

*Dr. Hodson.*—There are two or three points of suggestion which I have to offer just here, and, though separate in themselves, are all centred about the law of capillarity, a law of which I take advantage in so many directions and find so valuable that I had purposed making a separate paper on the subject. In the first place, I make sure of never pushing the septic contents of root-canals through the foramen, by alternately filling them with water and antiseptics and drawing them down and out again until the canal is clean. This I do by means of bibulous paper root-points, which I conceived for this purpose and which I gave to the profession in a paper read before the Odontological Society some twenty years ago. The idea being that while the paper point will penetrate to the full depth of a root-canal nearly as well as a broach, it will pull down the liquid contents and not push them ahead, like cotton on a broach.

Similarly, after the root-canals have been brought into a proper condition to fill, I use this same capillarity to even better purpose; so much so, indeed, that I am most confident that my canal-filling reaches wherever the end of the broach has gone. The root having been made perfectly dry (and it is astonishing how dry the canal can be made by letting the finely twisted paper points reaching to the end of the root lie half a minute before removing, then following with another, etc.), flood the canal with chloroform to wet the porous dentine with the menstruum of the chloro-percha which is to follow; then having wetted the whole surface of a very fine broach with thin chloro-percha, place it quickly in the canal to the end and as quickly touch it at the orifice of the canal with a drop of thin chloro-percha,—one can see it jump. Following the chloro-percha on the broach, a tiny working of the broach around and up and down will make assurance doubly sure of its adhesion to the walls. Then the solid and cold gutta-percha canal point, having been previously attached to the end of a root-canal plugger, is very carefully pointed at the entrance and quickly and gently carried snugly home. By this procedure the end of the gutta-percha point is never turned, but goes as a solid point practically to the end of the root, the preceding chloro-percha but serving to plug up the mouths of the tubuli, following as it does the penetrative soaking of the dentine with chloroform and making perfect the fitting of the solid gutta-percha point, the real canal filling.

In fine, my aim and expectation is to have at the end of the operation not much more chloro-percha remaining in my root than there would be of glue in a properly glued wooden joint.

*Dr. G. S. Allan.*—I would take exception to the term "capillary attraction" used by Dr. Hodson. Capillary attraction, of itself, could not to any extent draw a fluid into a tube, however fine, that was closed at one end.

*Dr. Hodson.*—I only offer a method for *filling* whenever the root is ready, a method I cannot but feel makes possible, even sure, the filling of roots to their ends with gutta-percha. As to tortuous roots, if I were only as certain, though employing every known means as I do,—sulphuric acid for opening and enlarging the canals and burning out the organic contents, the employment of germicides and disinfectants, hot air, etc.,—I say, if I were only as certain of getting these tortuous canals clean as I am of perfectly filling them by means of this capillary attraction acting along the finely coated broach, reaching loosely to the end of the root, I should feel very comfortable. I may add that this method applied to oxychloride of zinc is equally effective, substituting the chloride of zinc instead of chloroform for the preliminary wetting of the interior of the root, and is, indeed, the material with which I years ago first used the method. Dr. Allan remarks that capillary attraction does not act in a tube unless both ends are open. Quite true, but I do not use the tube; I use the coated broach laid along inside the tube (the root-canal). Granted that the broach is sufficiently fine and so thinly coated as not to fit tightly in this tube, and the interior surface being wetted with the menstruum of the filling-material, capillary attraction will surely carry such material to wherever the end of the broach may reach. Once the inside is thinly coated, it is followed by the solid gutta-percha, as before mentioned. That, I thought, Dr. Allan understood.

*Dr. G. S. Allan.*—Dr. Hodson's explanation does not prove his point to my mind; in fact, disproves it. The doctor uses a mechanical force to push his fluid into the root and fill it and close the foramen, and capillary attraction plays only a slight part in the operation. The walls of the pulp-canal are capable of being wetted,—if I may use the term,—and the doctor, by means of his broach, or broach wrapped with a fibre or so of cotton, brings the fluid into direct contact with the walls of the root and at the same time forces the contained air out. Capillary attraction to be operative in such a case requires a very fine tube and open at both ends. The chloro-percha will go just as far and fill just as fine and crooked a canal as the broach carries it, and practically no farther. Of course, you will understand that the chloro-percha brought—pressed—into contact with the walls of the pulp-canal clings to them by the law of capillary attraction, but that part of Dr. Hodson's statement has not been questioned.

*Dr. Kasson C. Gibson.*—It is claimed that it is necessary to thoroughly sterilize the roots of septic teeth before filling. I do not believe a tooth can be thoroughly sterilized with antiseptics. What assurance have you that a tooth can be sterilized? I use Evans's root dryer as well as antiseptics, relying more on heat. When the tooth is favorable for filling, as a rule, I fill the root-canal with zinc oxychloride. In the treatment of abscessed teeth I have not always met with success. From a surgical stand-point a large percentage of abscessed teeth should not be treated, but extracted.

*Dr. Onderdonk.*—I was in hopes, Mr. President, that the discussion would follow Dr. Gibson's suggestions. The points I wished brought forward were,—(1) the aseptic condition, how secured and how maintained; (2) what is the best permanent antiseptic root-filling?

How are we to know when the root is thoroughly sterilized? I suggested making cultures and the use of the microscope as possible means to that end. I wish some one more conversant had said something on this subject.

There are three principal sources of infection which should be taken into consideration,—the cavity in the tooth, the apical foramen, and the dentinal tubuli. The closing of these last I believe to be the weakest point in most filling-materials.

If chloro-percha is used, it will shrink in proportion to the bulk of chloroform in the solution. It will not adhere to the tooth-substance nor will it close the foramen. Oxychloride of zinc ceases to be an antiseptic on crystallization, and is soluble, thereby admitting infection at the apical foramen.

The method I suggested in the paper—*i.e.*, iodoform in ether, a varnish of rosin in chloroform, and gutta-percha points—seems to me to represent the most scientific root-filling. By this method, having the cavity rendered antiseptic by hot air, hot points, etc., the iodoform in ether is introduced, and the porous tooth-substance must absorb some of the iodoform. This is sealed in by the rosin varnish. The apical foramen is closed by the gutta-percha points with iodoform ahead of them, and the points solidly fill the canal. The cavity in the tooth is then closed with a suitable filling and the three sources of infection are taken care of.

*Dr. A. L. Swift.*—Referring to Dr. Onderdonk's objection to oxychloride as a root-filling, I have filled roots for more than fifteen years almost exclusively with oxychloride, and am fully convinced that when used at the proper consistency zinc chloride finds its way into the tubuli, especially when only non-coagulants have been used, and consequently all antiseptic action does not cease as soon as the crystallization takes place.

*Dr. Leroy.*—I wish to call attention to the remark that gutta-percha thoroughly dissolved in chloroform (chloro-percha) and placed in a root-canal in time occupies less space than it did at first, which phenomenon we know to be due to shrinkage. I would like to say that gutta-percha dissolved in eucalyptus oil (eucalypto-percha) does not contract but remains the same, the eucalyptus oil even penetrating into the dentinal tubuli. I have found that to be the best material, and have used it for a long time (seven years or so).

Adjourned.

FRED. L. BOGUE, M.D., D.D.S.,
*Editor The New York Institute of Stomatology.*

